# ICOS and ICOS ligand: expression patterns and outcomes in oncology patients

**DOI:** 10.1177/17588359251330514

**Published:** 2025-04-24

**Authors:** Mina Nikanjam, Shumei Kato, Daisuke Nishizaki, Donald A. Barkauskas, Sarabjot Pabla, Mary K. Nesline, Jeffrey M. Conroy, Aung Naing, Razelle Kurzrock

**Affiliations:** Division of Hematology–Oncology, University of California San Diego, 1200 Garden View Road, La Jolla, CA 92024, USA; Division of Hematology–Oncology, University of California San Diego, La Jolla, CA, USA; Division of Hematology–Oncology, University of California San Diego, La Jolla, CA, USA; Biostatistics Division, Department of Population and Public Health Sciences, Keck School of Medicine, University of Southern California, Los Angeles, CA, USA; Labcorp Oncology, Buffalo, NY, USA; Labcorp Oncology, Buffalo, NY, USA; Labcorp Oncology, Buffalo, NY, USA; Department of Investigational Cancer Therapeutics, MD Anderson Cancer Center, Houston, CA, USA; Medical College of Wisconsin Cancer Center, Milwaukee, WI, USA; WIN Consortium, Chevilly-Larue, France

**Keywords:** ICOS, ICOS ligand, immune checkpoint, immunotherapy

## Abstract

**Background:**

Inducible T-cell co-stimulator (ICOS) and its ligand (ICOSL) form a complex, two-faced immune machinery that can lead to both immune stimulation and inhibition.

**Objective:**

We explored ICOS transcriptomic expression patterns and their relationship with other checkpoints and with outcomes in patients with advanced/metastatic cancers.

**Design:**

This was a retrospective cohort study.

**Methods:**

RNA expression for ICOS and other immune checkpoints was quantified by RNA sequencing and stratified by rank values into high (75–100 percentiles) and low (0–24 percentiles). Fischer’s exact tests were used for univariate analyses to evaluate independent predictors of ICOS high and logistic regression was used for multivariate analyses. Progression-free survival (PFS) and overall survival (OS) for ICOS high versus not high expression were evaluated using the log-rank test (Kaplan–Meier analysis) and Cox proportional hazards.

**Results:**

High ICOS (⩾75 percentile RNA rank) was present in 14% of 514 cancers and independently associated with high PD-1 (*p* = 0.025), PD-L1 (*p* < 0.0001), and CTLA-4 RNA expression (*p* < 0.0001) and with patients not having colorectal cancer (*p* = 0.0009; multivariate analysis). Patterns of ICOS and ICOSL expression varied between and within tumor types. For 217 patients receiving immune checkpoint inhibitors (ICIs), there were no significant differences in PFS or OS between patients with ICOS high versus not-high expression (multivariate analysis). In 272 immunotherapy-naïve patients, OS was also similar between patients with ICOS high versus not-high expression (*p* = 0.91).

**Conclusion:**

High ICOS expression was not a prognostic marker and did not independently predict outcomes after ICIs. Variable expression of ICOS/ICOSL between tumors and association of high ICOS with high PD-1, PD-L1, and CTLA-4 suggest that individual tumor immunomic analysis may be required for optimized patient selection in clinical trials targeting the ICOS/ICOSL system, especially when given in combination with ICIs.

**Trial registration:**

UCSD_PREDICT, NCT02478931.

## Introduction

Inducible T-cell co-stimulator (ICOS; CD278) and its unique ligand (ICOSL) form a complex and two-faced immune machinery, which contributes to both anti-tumor responses/immune stimulation and potentiation of immunosuppression.^1–4^ ICOSL is constitutively expressed by antigen-presenting cells including B cells, macrophages, and dendritic cells, along with many somatic cells, while ICOS is only expressed on a small fraction of resting T cells at low levels after activation.^1^ ICOS on T cells interacts with ICOSL on antigen-presenting cells. ICOS co-stimulation results in cytokine production (IL-4, IL-5, IL-6, IL-10, IL-13, and IL-21) in addition to interferon-gamma and TNF-alpha^1^; however, it does not provide efficient IL-2 production by activated T cells.^1^ Co-simulation leads to effector T-cell proliferation and survival through recruitment of phosphatidylinositol 3-kinase, memory formation, cooperation of T and B cells, development of Th1, Th2, and Th17 cells along with an increase in CD8+ cytotoxic T lymphocytes, and the differentiation of follicular helper T cells.^1^ ICOS also regulates effector T-cell subset differentiation and helps establish CD8+ tissue-resident memory T-cells.^5^ Simultaneously, the binding of ICOSL on antigen-presenting cells or tumor cells to ICOS on regulatory T-cells leads to an increase in IL-10 and TGF-beta along with regulatory T-cell proliferation.^1,6–11^

ICOS/ICOSL pathway activation has been observed in multiple cancer types^6^; thus, ICOS-modulating agents are under development in combination with Food and Drug Administration (FDA)-approved immune checkpoint inhibitors (ICIs) to improve responses and outcomes in advanced and metastatic cancers.^1^ ICOS-targeting therapies are being studied in combination with CTLA-4, PD-1, or PD-L1 antibodies.^1,12–19^ Given that ICOS/ICOSL pathway activation can both stimulate and inhibit the immune system, developing appropriate targets and therapeutics can be challenging.

Additional information regarding ICOS expression and its relationship to other immunoregulatory molecules would be helpful to better develop ICOS immune modulators as novel therapeutics. The current study describes ICOS expression patterns across and within tumor types and in relation to other clinically relevant checkpoints as well as with clinical outcomes.

## Materials and methods

### Patients

Data were assessed from 514 consecutive patients with a variety of advanced solid tumors seen at the University of California San Diego Center for Personalized Therapy who had RNA expression testing performed. RNA expression levels of ICOS, ICOS ligand, PD-1, PD-L1, LAG-3, and CTLA-4 from tumor specimens were analyzed at Labcorp Oncology (Buffalo Lab) (https://oncology.labcorp.com/), a Clinical Laboratory Improvement Amendments-licensed and College of American Pathologist-accredited clinical laboratory. Tumor mutational burden (TMB) was also evaluated. Each patient’s age, gender, and tumor histology were collected from the electronic medical record. For patients with more than one tumor sample collected on different dates, the earlier sample was used for the analysis. Patients in the study were a retrospective cohort and subset of the University of California San Diego PREDICT protocol (Study of Personalized Cancer Therapy to Determine Response and Toxicity, UCSD_PREDICT, NCT02478931; https://clinicaltrials.gov/study/NCT02478931; PREDICT study registration: September 5, 2013, dates of recruitment of patients for subset analysis: July 2017–November 2020). The reporting of this study conforms to the Strengthening the Reporting of Observational Studies in Epidemiology (STROBE) Statement: guidelines for reporting observational studies statement^20^ (STROBE Checklist in the Supplemental Material).

### Sampling of tissue and analysis of cancer immunity markers

After collection, tumors were provided as formalin-fixed, paraffin-embedded (FFPE), and evaluated via RNA sequence at the OmniSeq laboratory. RNA was extracted from FFPE using a truXTRAC FFPE extraction kit (Covaris, Inc., Woburn, MA, USA). After purification, RNA was dissolved in 50 µL water, and the yield was determined via Quant-iT RNA HS assay (Thermo Fisher Scientific, Waltham, MA, USA). The pre-defined titer of 10 ng RNA was referred to as the acceptance criteria for appropriate library preparation. Absolute reading of the RNA sequence was completed using Torrent Suite’s plugin immuneResponseRNA (v5.2.0.0, ThermoFisher Scientific, Waltham, MA) 34, and RNA expression of 397 distinct genes was determined.

Transcript abundance was normalized to internal housekeeping gene profiles, ranked (0–100 percentile) in a standardized manner to a reference population of 735 tumors which included 35 separate tumor types, and RNA expression profiles were stratified by rank values into “High” (75–100 percentile), “Intermediate” (25–74 percentile), and “Low” (0–24 percentile).

To determine TMB, genomic DNA was extracted from FFPE tumor samples that had greater than 30% neoplastic nuclei using the truXTRAC FFPE extraction kit (Covaris, Inc.) with 10 ng DNA input for library preparation. DNA Libraries were prepared using the Ion AmpliSeq targeted sequencing chemistry and the Comprehensive Cancer Panel, followed by enrichment and template preparation with the Ion Chef system, and then sequencing on the Ion S5XL 540 chip (Thermo Fisher Scientific). After removing germline variants, synonymous variants, indels, and single nucleotide variants with less than 5% variant allele fraction, TMB was reported as eligible mutations per qualified panel size (Mutations/Mb).

### Statistics/data analysis

Fischer’s exact tests were utilized for univariate analyses of high ICOS expression. Age, gender, cancer type, high LAG-3, high TMB, high CTLA-4, high PD-1, and high PD-L1 were evaluated to determine which were independent predictors of ICOS high (see also Supplemental Methods). The relationships between ICOS high and ICOS ligand high or low expression were also assessed. Variables that were significant in univariate analysis (*p* ⩽ 0.05) were included in multivariate analysis via logistic regression. Linear regression was utilized to compare ICOS RNA expression to variables significant in the multivariate screen and a *p*-value ⩽0.05 was considered significant. All analyses were performed using SAS v. 9.4, SAS Institute, Cary, North Carolina.

Progression-free survival (PFS) and overall survival (OS) from the start of treatment with ICIs were compared for patients by ICOS high versus not high expression using the log-rank test (Kaplan–Meier analysis) and Cox proportional hazards regression. OS for patients not receiving ICIs from the time of advanced or metastatic disease was compared between patients by ICOS high versus not high expression using the log-rank test (Kaplan–Meier analysis) and Cox proportional hazards regression. If the log-rank test for ICOS high versus not high was significant, then ICOS high versus not high was tested along with covariates significant in Fischer’s exact test in a multivariate Cox proportional hazards regression. Patients who had not progressed or died at the time of the last follow-up were censored at that date. Patients missing detailed clinical data such as date of advanced/metastatic disease or treatment history were excluded from the survival analyses. All statistical analyses were verified by our biostatistician (D.A.B.). SAS v. 9.4 was used and *p*-values <0.05 were considered significant.

### Data availability

The datasets used and/or analyzed during the current study are available from the corresponding authors upon reasonable request.

## Results

### Patient characteristics

Data from 514 patients with a variety of cancer types were utilized for the analysis (Table 1).^21–29^ The median age was 60.8 years. Men represented 40% of patients (*N* = 204) and women represented 60% (*N* = 310). The most common cancer types evaluated for ICOS were colorectal cancer (*N* = 140), pancreatic cancer (*N* = 55), breast cancer (*N* = 49), and ovarian cancer (*N* = 43; Table 1). High TMB (⩾10 mutations/Mb) was seen in 7% (*n* = 33/450) of evaluable patients. High PD-1 was seen in 18% (*n* = 93/514), high PD-L1 in 13% (*n* = 67/514), high CTLA-4 in 17% (*n* = 87/514), and high LAG-3 in 23% (*n* = 116/514 patients; all ⩾75 percentile RNA rank). Altogether, 217 patients received an ICI; 197 of these patients (91%) received an anti-PD-1 agent. Two-hundred seventy-two patients did not receive immune checkpoint inhibitors and 25 did not have sufficient clinical information to be included in the clinical outcomes analysis. The average duration of follow-up was 30 months.

**Table 1.** Demographics for all patients and those receiving immune checkpoint inhibitors.

All patients (*N* = 514)		
Gender		
Male		204 (40%)
Female		310 (60%)
Age (years)		
Median (25%–75% interquartile range)	60.8 (50.5–69.5)	
	Total	*N* (%)
Most common tumor types		
Colorectal	514	140 (27%)
Pancreatic	514	55 (11%)
Breast	514	49 (10%)
Ovarian	514	43 (8%)
Expression markers^b^		
High ICOS	514	70 (14%)
High TMB (⩾10 mutations/Mb)	450^c^	33 (7%)
High PD-1	514	93 (18%)
High PD-L1	514	67 (13%)
High CTLA-4^a^	514	87 (17%)
High LAG-3	514	116 (23%)
Patients receiving immune checkpoint inhibitors (*N* = 217)		
Gender		
Male		95 (44%)
Female		122 (56%)
Age (years)		
Median (25%–75% interquartile range)	61.9 (51.7–70.4)	
	Total	*N* (%)
Most common tumor types		
Colorectal	217	54 (25%)
Pancreatic	217	19 (9%)
Breast	217	18 (8%)
Ovarian	217	16 (7%)
All patients (*N* = 514)		
Immune checkpoint inhibitors		
Total receiving anti-PD-1 agent	217	197 (91%)
Pembrolizumab	217	128 (59%)
Nivolumab	217	51 (24%)
Ipilimumab/nivolumab	217	14 (6%)
Atezolizumab	217	10 (5%)
Other immunotherapies^a^	217	14 (6%)
RNA expression^b^		
High ICOS	217	30 (14%)

This database has been previously reported.^21–29^

a Other immunotherapies: duravalumab (*n* = 6), ipilimumab (*n* = 2), avelumab (*n* = 2), ipilimumab/pembrolizumab (*n* = 2), cemiplimab (*n* = 1), and dostarlimab (*n* = 1).

b High CTLA-4, high PD-1, high PD-L1, high ICOS, high ICOS-L, high LAG-3 ⩾75 percentile RNA rank; Low ICOS, ICOS-L 0–24 percentile RNA rank.

c TMB assessment was only available in 450 patients.

ICOS, inducible T-cell co-stimulator; ICOSL, inducible T-cell co-stimulator ligand; TMB, tumor mutational burden.

#### ICOS and ICOSL RNA expression was variable between tumors

**Figure 1.**
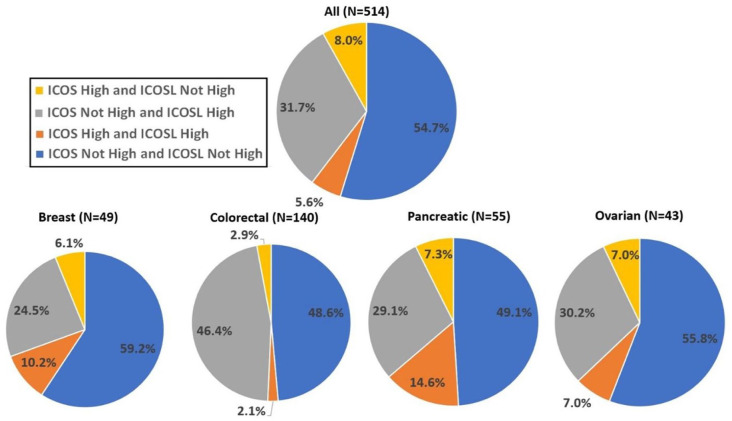
Proportion (%) of patients with different combinations of ICOS and ICOSL high and not high (low-moderate) expression for all malignancies and the most common cancer types. High, ⩾75 percentile RNA rank; not high, 0–74 percentile RNA rank. ICOS, inducible T-cell co-stimulator; ICOSL, inducible T-cell co-stimulator ligand.

The proportion of patients with different combinations of ICOS and ICOSL high (≥75th percentile RNA rank) and not high (<75th percentile RNA rank) are shown in Figure 1. A minority of patients co-expressed ICOS and ICOSL high (5.6%), whereas 31.7% co-expressed ICOSL high with ICOS not high, and a minority (8.0%) expressed ICOS high with ICOSL not high. For the most common cancer types in the current study, ICOS and ICOSL high were co-expressed in 2.0% (colorectal) to 14.6% (pancreatic), whereas ICOSL high was co-expressed with ICOS not high in 29.1% (pancreatic) to 46.4% (colorectal) of patients. ICOS high was co-expressed with ICOSL not high in 2.9% (colorectal) to 7.3% (pancreatic) of patients.

#### High ICOS RNA expression was most common in esophageal and pancreatic cancer and the least common in colorectal cancer

**Figure 2.**
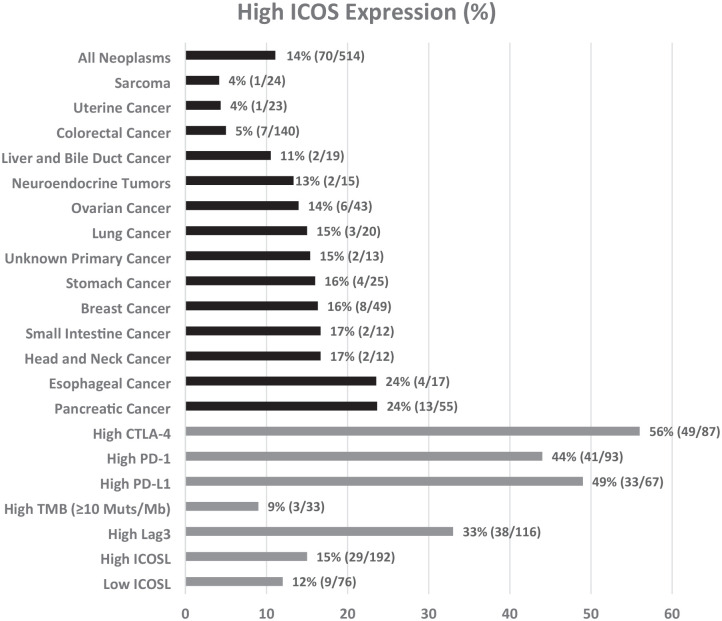
High ICOS expression according to cancer type and according to high levels of other immunomodulatory markers (⩾75 percentile RNA rank). Only cancers with at least 10 samples were included in the figure. Overall, 70 of 514 cancers (~14%) had high ICOS RNA expression. Forty-nine of 87 cancers (56%) with high CTLA-4 had high ICOS. High TMB, tumor mutational burden ⩾10 mutations/Mb. ICOS, inducible T-cell co-stimulator; ICOSL, inducible T-cell co-stimulator ligand.

High ICOS expression (⩾75 percentile RNA rank) was observed in 70 of 514 patients (14%). High ICOS expression was seen most commonly in esophageal cancer (24%; 4/17) and pancreatic cancer (24%; 13/55) and least commonly observed in sarcoma (4%; 1/24; Figure 2, Table 2, and Supplemental Figure 1(A)). However, expression was variable even within tumor types, with 42% of pancreatic cancer showing low levels (<25th RNA percentile rank) of ICOS expression (Supplemental Table 1 and Supplemental Figure 1(B)). Multivariate analysis demonstrated that high ICOS RNA levels were independently associated with patients not having colorectal cancer (*p* = 0.0009; Table 2).

**Table 2.** Factors associated with high ICOS expression.

Demographics/Expression Markers	High ICOS^a^ (*N* = 70)	Odds ratio (95% CI)	Univariate *p* value^d^	Multivariate *p* value^b,d^	Comment
Men (*N* = 204) Women (*N* = 310)	Men (*N* = 26/204, 12.8%) Women (*N* = 44/310, 14.2%)	0.88 (0.52–1.49)	0.69		
Age ⩾median (61 years) (*N* = 256) and <median (*N* = 258)	Age ⩾median (*N* = 37/256, 14.5%) Age <median (*N* = 33/258, 12.8%)	1.15 (0.69–1.91)	0.61		
Tumor types					
Colorectal (*N* = 140) Non-colorectal (*N* = 374)	Colorectal (*N* = 7/140, 5.0%) Non-colorectal (*N* = 63/374, 16.8%)	0.26 (0.12–0.58)	**0.0003**	**0.0009**	High ICOS is independently associated with non-colorectal cancer and with high PD-1, high PD-L1, and high CTLA-4 RNA expression
Pancreatic (*N* = 55) Non-pancreatic (*N* = 459)	Pancreatic (*N* = 12/55, 21.8%) Non-pancreatic (*N* = 58/459, 12.6%)	1.92 (0.96–3.87)	0.09		
Breast (*N* = 49) Non-breast (*n* = 465)	Breast (*N* = 8/49, 16.3%) Non-breast (*N* = 62/465, 13.3%)	1.27 (0.57–2.83)	0.52		
Ovarian (*N* = 43) Non-ovarian (*N* = 471)	Ovarian (*N* = 6/43, 14.0%) Non-ovarian (*N* = 64/471, 13.6%)	1.03 (0.42–2.54)	1.00		
High TMB (*N* = 33)^c^ Not high TMB (*N* = 417)	High TMB (*N* = 3/33, 9.1%) Not high TMB (*N* = 50/417, 12.0%)	0.73 (0.22–2.49)	0.78		
High PD-1 (*N* = 93)^a^ Not high PD-1 (*N* = 421)	High PD-1 (*N* = 41/93, 44.1%) Not high PD-1 (*N* = 29/421, 6.9%)	10.7 (6.1–18.6)	**<0.0001**	**0.025**	
High PD-L1 (*N* = 67)^a^ Not high PD-L1 (*N* = 447)	High PD-L1 (*N* = 33/67, 49.3%) Not high PD-L1 (*N* = 37/447, 8.3%)	10.8 (6.0–19.3)	**<0.0001**	**<0.0001**	
High CTLA-4 (*N* = 87)^a^ Not High CTLA-4 (*N* = 427)	High CTLA-4 (*N* = 49/87, 56.3%) Not high CTLA-4 (*N* = 21/427, 4.9%)	24.9 (13.5–45.9)	**<0.0001**	**<0.0001**	
High LAG-3 (*N* = 116)^a^ Not High LAG-3 (*N* = 398)	High LAG-3 (*N* = 38/116, 32.8%) Not high LAG-3 (*N* = 32/398, 8.0%)	5.6 (3.3–9.5)	**<0.0001**	0.50	
High ICOSL (*N* = 192)^a^ Not High ICOSL (*N* = 322)	High ICOSL (*N* = 29/192, 15.1%) Not high ICOSL (*N* = 41/322, 12.7%)	1.22 (0.73–2.04)	0.51		
Low ICOSL (*N* = 76)^a^ Not Low ICOSL (*N* = 438)	Low ICOSL (*N* = 9/76, 11.8%) Not low ICOSL (*N* = 61/438, 13.9%)	0.83 (0.39–1.75)	0.72		

a High CTLA-4, high PD-1, high PD-L1, high LAG-3, high ICOSL ⩾75 percentile RNA rank; low ICOSL 0–24 percentile RNA rank; not high CTLA-4, not high PD-1, not high PD-L1, not high LAG-3, not high ICOSL <75 percentile RNA rank; not low ICOSL >24 percentile RNA rank.

b *p* Values that were significant (*p* ⩽ 0.05) were included in multivariate analysis.

c High TMB, ⩾10 mutations/Mb; not high TMB <10 mutations/Mb.

d *p* Values that were significant (*p* ≤ 0.05) are denoted in bold.

ICOS, inducible T-cell co-stimulator; ICOSL, inducible T-cell co-stimulator ligand; TMB, tumor mutational burden.

#### High ICOSL expression was independently associated with colorectal cancer

High ICOSL expression was variable between cancers (Supplemental Figure 1(A)). Altogether, 37% of patients (192/514) had high ICOSL RNA levels. Interestingly, in contrast to the low numbers of colorectal cancer patients with high ICOS, high ICOSL was independently associated with colorectal cancer (and was found in 49% (68/140) colorectal tumors; Supplemental Table 2). Low ICOSL was independently associated with not having colorectal cancer and with low ICOS (Supplemental Table 3).

#### High ICOS RNA expression correlated with high RNA expression of checkpoints CTLA-4, PD-1, and PD-L1

**Figure 3.**
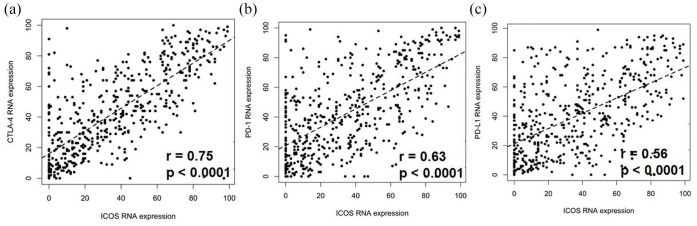
A linear relationship between ICOS RNA expression versus (a) CTLA-4, (b) PD-1, and (c) PD-L1 RNA expression. Pearson correlation coefficient is represented by r on graphs. ICOS, inducible T-cell co-stimulator.

High ICOS RNA expression (>75th percentile RNA rank) was commonly seen in tumors with high CTLA-4 (56%; 49/87), high PD-1 (44%; 41/93), and high PD-L1 (49%, 33/67) transcript expression. Multivariate analysis demonstrated that high ICOS RNA levels were independently associated with tumors with high PD-1 RNA expression (*p* = 0.025), tumors with high PD-L1 RNA expression (*p* < 0.0001), and tumors with high CTLA-4 RNA expression (*p* < 0.0001; Table 2). Significant linear relationships between ICOS and CTLA-4 RNA (*p* < 0.0001, *r* = 0.75), PD-1 RNA (*p* < 0.0001, *r* = 0.63), and PD-L1 RNA (*p* < 0.0001, *r* = 0.56) expression were also observed (Figure 3).

Low ICOS (<25th percentile RNA rank; found in 44% of 514 cancers) was independently associated with not-high (<75th percentile RNA rank) PD-1, not-high PD-L1, not-high CTLA-4, and low ICOS ligand (Supplemental Table 1 and Supplemental Figure 1(B)).

#### High ICOS RNA levels predicted longer OS in univariate (but not in multivariate) analysis after treatment with ICIs

**Figure 4.**
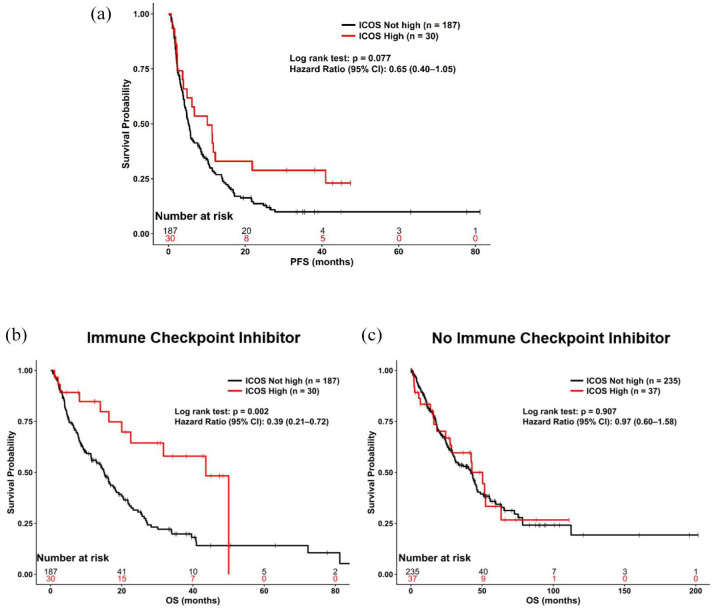
(a) PFS from the start date of first immunotherapy for patients who received immunotherapy (*N* = 217) stratified by ICOS high (ligand ⩾75 percentile RNA rank) versus not high. OS stratified by ICOS high (ligand ⩾75 percentile RNA rank) versus not high. (b) Received immunotherapy (*N* = 217) from the start date of first immunotherapy. OS result was not significant in the multivariate screen (*p* = 0.28; hazard ratio (95% confidence interval): 0.63 (0.27–1.45) (tested with colorectal cancer disease type, PD-1 high vs not high, PD-L1 high vs not high, and CTLA-4 high vs not high). (c) Did not receive immunotherapy (*N* = 272) from the date of diagnosis of advanced or metastatic disease. High ICOS ⩾75 percentile RNA rank. ICOS, inducible T-cell co-stimulator; OS, overall survival; PFS, progression-free survival.

A total of 217 patients with advanced/metastatic disease received an ICI (Table 1). The most common ICI administered was pembrolizumab. PFS was similar between patients with ICOS high versus not high expression (*p* = 0.08; hazard ratio (95% confidence interval): 0.65 (0.40–1.05); Figure 4(a)). OS was longer for ICOS high as compared to ICOS not high (*p* = 0.002; hazard ratio (95% confidence interval): 0.39 (0.21–0.73); Figure 4(b); from the start date of immunotherapy); however, this was not significant in multivariate analysis (*p* = 0.28; hazard ratio (95% confidence interval): 0.63 (0.27–1.45); when tested with colorectal cancer disease type, PD-1 high vs not high, PD-L1 high vs not high, and CTLA-4 high vs not high). Of the patients included in the analysis, 85 (39%) had not died at the time of the last follow-up and were censored at that date.

Other permutations were also examined. PFS for patients with ICOSL high versus not high with ICOS low versus not low or with ICOSL low versus not low did not differ significantly after immunotherapy (Supplemental Figure 2). ICOSL levels did not correlate with OS after immunotherapy (Supplemental Figure 3).

#### High ICOS RNA levels did not correlate with prognosis in patients who were immunotherapy naïve

A total of 272 subjects with advanced/metastatic disease did not receive ICIs at any time. OS was similar between patients with ICOS high versus not high expression (*p* = 0.91; hazard ratio (95% confidence interval): 0.97 (0.60–1.58); Figure 4(c)). Of the patients included in the analysis 138 (51%) had not died at the time of the last follow-up and were censored at that date.

Similarly, ICOSL high versus not high, ICOS low versus not low, and ICOSL low versus not low were not prognostic factors as they did not correlate with OS in immunotherapy-naïve patients (Supplemental Figure 4).

## Discussion

ICOS and ICOSL are part of an important immune checkpoint pathway, which is being explored in clinical trials as a novel immunotherapy target. However, the interaction between ICOS and ICOSL and its effects on the immune system are complicated. The ICOS/ICOSL pathway can contribute to both immune stimulation and immunosuppression. Thus, it is critical to better understand relationships between ICOS with other immune checkpoints and tumor types to develop effective novel therapeutics targeting this pathway.

The current study evaluated ICOS and ICOSL RNA expression levels in relationship to other ICIs and cancer types. High ICOS was expressed in a minority of cancers (14%) and was most commonly seen in esophageal (24%) and pancreatic (22%) cancers. High expression of ICOS RNA was associated with high PD-1, high PD-L1, high CTLA-4, and non-colorectal cancers. For patients receiving ICIs, there was no difference in PFS (*p* = 0.08) or OS (*p* = 0.28 in multivariate screen) for patients whose tumors had high ICOS as compared to not high expression. High ICOSL was expressed in a minority of cancers (37%). High ICOSL RNA was associated with colorectal cancer, not-high PD-L1, and with high CTLA-4. For patients receiving ICIs, there was no difference in PFS (*p* = 0.55) or OS (*p* = 0.97) for patients whose tumors had high ICOSL as compared to not high expression. A minority of patients co-expressed ICOS and ICOSL high (5.6%), whereas 31.7% co-expressed ICOSL high with ICOS not high, and a minority (8.0%) expressed ICOS high with ICOSL not high. For patients not receiving ICIs, there was no significant difference in survival for tumors with ICOS (*p* = 0.91) or ICOSL (*p* = 0.25) high versus not high expression. Thus, ICOS and ICOSL high expression alone were not prognostic markers for metastatic cancers and do not predict better outcomes following ICI administration.

There are a number of ICOS/ICOSL pathway-targeting agents in clinical trials (Supplemental Table 4). The majority of oncology clinical trials to date have focused on the development of ICOS agonists with only one study exploring an ICOS antagonist. Responses rates to ICOS/ICOSL-directed therapies have been limited in most clinical trials. JTX2011/vopratelimab, an ICOS agonist, had a response rate of 1.4% for monotherapy and 2.3% when combined with PD-1-directed therapy in solid tumors.^12^ JTX2011/vopratelimab was also evaluated in non-small cell lung cancer with a biomarker-selected approach with a response rate of 27.3%.^14^ GSK3359609/feladilimab, an ICOS agonist, had a 26% response rate in combination with pembrolizumab in head and neck cancer^15^ and a 22% response rate in combination with pembrolizumab in urothelial cancers (8% single agent response rate).^16^ XmAb23104 is a bispecific antibody that serves both as an ICOS agonist and a PD-1 inhibitor which had a single agent response rate of 4.8% in solid tumors.^17^ MEDI-570, an ICOS antagonist, had a response rate of 44% in angioimmunoblastic T-cell lymphoma.^18^ KY1044/alomfilimab SAR445256, a dual mechanism molecule that binds with high affinity to ICOS promoting preferential depletion of Tregs with high ICOS and stimulates Teff with low ICOS, had a 5% response rate in advanced cancers.^19^

Thus, ICOS targeting agents appear to be more effective when given in combination therapy. In preclinical studies, ICOS agonists potentiated the effects of anti-CTLA-4 therapy and ICOS has been observed clinically to be upregulated with anti-CTLA-4 treatment; thus, combination therapy with ICOS agonists may increase efficacy over administrating anti-CTLA-4 alone.^1^ The association between high ICOS expression and high PD-1, high PD-L1, and high CTLA-4 expression also suggests that patients with high ICOS expression would likely benefit from combination therapy with ICOS-directed therapy and PD-1 and/or CTLA-4 inhibitors. It should be mentioned that ICOS, PD-1, and CTLA-4 are all T-cell activation markers, and thus, the observed correlation between these checkpoints may be due to higher T-cell activation. Also, PD-L1 is IFN-gamma-dependent; thus, its increased expression in tumors with potentially high T-cell activation is not surprising.

The ICOS/ICOSL system is complicated. It may be that ICOSL agonists might be most effective in the presence of high ICOS and low ICOSL. ICOS agonists might be most effective when ICOS is low. However, the cells on which ICOS and ICOSL are expressed may also be of paramount importance. A dual mechanism ICOS modulator such as KY1044^19^ serves as an agonist/depleter depending on the levels of ICOS on target cells. As such, it is a therapeutic option for tumors with high ICOS in Tregs but low ICOS in effectors and tumors in which ICOS is a tumor-associated antigen.^30,31^ It is likely contraindicated in tumors with higher ICOS expression in effector than Treg cells. In diseases such as T-cell lymphoma, attenuating the effects of ICOS stimulation may be important and, therefore, an ICOS antagonist may be appropriate.^31^

While CTLA-4 and PD-1 inhibitors have led to activation of the immune system and dramatic responses in many patients with immunologically driven tumors, there are a number of patients who fail to respond or eventually develop resistance to these therapies.^32^ One possibility to overcome resistance and improve responses is to exploit other immune checkpoints such as ICOS/ICOSL with combination therapeutic approaches.^33^ Given that a minority of cancers express high ICOS (14%) and high expression of PD-1, PD-L1, and CTLA-4 was 18%, 13%, and 17%, respectively, biomarker selected trials will be critical to find clinical benefit for combination studies. ICOS-modulating agents such as KY1044 have shown promise in activating “cold tumors” to increase T-cell infiltration and decrease Tregs, thereby creating a tumor microenvironment more amenable to an antitumor immune response.^30^ A prior study found that inhibition of ICOS signaling before PD-1 immunotherapy, thereby interrupting the intratumor CD8^+^ T cell and Treg crosstalk, can improve the efficacy of PD-1-directed therapy.^34^ Thus, ICOS-directed therapies may play an important role in circumventing immunotherapy resistance for combination therapy.

ICOS/ICOSL pathway activation has been observed in multiple cancer types including melanoma, myeloma, breast, ovarian, gastric, liver, and colorectal cancers.^35^ ICOS expression on Tregs after IL-2 therapy and the presence of Tregs in TILs has been associated with poor clinical outcomes in metastatic melanoma^36,37^; however, ICOS presence on other TILs has been associated with better prognosis.^38^ Elevated ICOS-positive Tregs in localized renal cell carcinoma were associated with poor prognosis.^39^ ICOS was also shown to be an important element in the persistence of CD4+ chimeric Ag receptor T cells which has been approved for several hematological malignancies and is in clinical trials for a wide variety of other malignancies.^40^ In colorectal cancer, ICOS has been associated with higher CTLA-4 and PD-1 expression in lymphocytes.^41^ This further speaks to the importance of targeting the ICOS/ICOSL pathway for combination immunotherapy approaches.

The current study has several limitations. We measured RNA expression levels and did not differentiate ICOS/ICOSL in Tregs versus CD4/CD8 cells. In future studies, it may be helpful to do single-cell RNAseq to better understand the web of interactions between immunostimulatory and immunosuppressive elements and the tumor. The current study had a relatively small sample size and represented diverse cancer types; thus, the relationships with individual tumor types could only be explored in the most common tumor types. We focused on relationships with CTLA-4, PD-1, PD-L1, and LAG-3 that were felt to be of the highest clinical relevance; however, other genes may be found that are co-expressed with ICOS/ICOSL that would benefit from clinical development and exploration.

## Conclusion

In summary, the ICOS/ICOSL pathway represents an important and complex immunomodulatory system, with both stimulatory and suppressive effects. ICOS/ICOSL is being explored as a target for therapeutics in combination immunotherapy approaches. Our study suggests that tumors with high ICOS will likely express higher PD-1, PD-L1, and CTLA-4 transcripts and thus an ICOS-directed therapy with a PD-1/PD-L1 inhibitor or CTLA-4-directed therapy may be of benefit. However, expression levels vary both between and within tumor types and hence individual immunomic analysis for patient selection might be needed. High ICOS expression was not an independent prognostic marker and did not independently predict outcomes after the administration of FDA-approved checkpoint inhibitors. The decision to utilize an ICOS agonist or antagonist will depend on the ICOS expression level as well as location.

## Supplemental Material

sj-docx-1-tam-10.1177_17588359251330514 – Supplemental material for ICOS and ICOS ligand: expression patterns and outcomes in oncology patients Supplemental material, sj-docx-1-tam-10.1177_17588359251330514 for ICOS and ICOS ligand: expression patterns and outcomes in oncology patients by Mina Nikanjam, Shumei Kato, Daisuke Nishizaki, Donald A. Barkauskas, Sarabjot Pabla, Mary K. Nesline, Jeffrey M. Conroy, Aung Naing and Razelle Kurzrock in Therapeutic Advances in Medical Oncology

sj-docx-2-tam-10.1177_17588359251330514 – Supplemental material for ICOS and ICOS ligand: expression patterns and outcomes in oncology patients Supplemental material, sj-docx-2-tam-10.1177_17588359251330514 for ICOS and ICOS ligand: expression patterns and outcomes in oncology patients by Mina Nikanjam, Shumei Kato, Daisuke Nishizaki, Donald A. Barkauskas, Sarabjot Pabla, Mary K. Nesline, Jeffrey M. Conroy, Aung Naing and Razelle Kurzrock in Therapeutic Advances in Medical Oncology

## Declarations

**Ethics approval and consent to participate:** Study of Personalized Cancer Treatment to Determine Response and Toxicity, UCSD PREDICT, (NCT02478931) was carried out in conformity with the regulations of the UCSD Institutional Review Board, and any experimental interventions for which patients provided consent.

**Consent for publication:** Not applicable.

**Availability of data and materials:** The datasets used and/or analyzed during the current study are available from the corresponding authors upon reasonable request.
