# Supplementary material for: ICOS and ICOS ligand: expression patterns and outcomes in oncology patients
Source: Ther Adv Med Oncol. 2025 Apr 24;17:17588359251330514. doi: 10.1177/17588359251330514 (PMC12035295; doi:10.1177/17588359251330514)
Supplement: sj-docx-2-tam-10.1177_17588359251330514 – Supplemental material for ICOS and ICOS ligand: expression patterns and outcomes in oncology patients [file sj-docx-2-tam-10.1177_17588359251330514.docx]

**Supplemental Materials:**

**SUPPLEMENTAL METHODS**

Fischer’s exact tests were utilized for univariate analyses of both high and low ICOS and ICOS ligand expression (high ICOS, high ICOS-L ≥ 75 percentile RNA rank; low ICOS, ICOS ligand 0-24 percentile RNA rank). Age, gender, cancer type, high LAG-3, high TMB, high CTLA4, high PD-1, and high PD-L1 were evaluated to determine which were independent predictors of ICOS (high vs. not high, low vs. not low) and ICOS ligand (high vs. not high, low vs. not low). The relationships between ICOS and ICOS ligand high or low expression were also assessed. Variables that were significant in univariate analysis (p ≤ 0.05) were included in multivariate analysis via logistic regression. All analyses were performed using SAS v. 9.4.

Progression-free survival (PFS) and overall survival (OS) from the start of treatment with immune checkpoint inhibitors was compared for patients by ICOS (high vs. not high, low vs. not low) and ICOS ligand (high vs. not high, low vs. not low) expression using the log-rank test (Kaplan-Meier analysis) and Cox proportional hazards regression. OS for patients not receiving immune checkpoint inhibitors from time of advanced or metastatic disease was compared between patients by ICOS (high vs. not high, low vs. not low) and ICOS ligand (high vs. not high, low vs. not low) expression using the log-rank test (Kaplan-Meier analysis) and Cox proportional hazards regression. Patients who had not progressed or died at the time of last follow-up were censored at that date. All statistical analyses were verified by our biostatistician (DAB). SAS v. 9.4 was used and p-values <0.05 were considered significant.

**Supplemental Table 1. Factors associated with low ICOS (<25^th^ percentile RNA rank) expression**

|  | **Low ICOS* (N=226)** | **Odds ratio (95% CI)** | **Univariate P value** | **Multivariate P value**** | **Comment** |
| --- | --- | --- | --- | --- | --- |
| Men (N=204)  Women (N=310) | Men (N=84/204, 41.2%)  Women (N=142/310, 45.8%) | 0.83 (0.58-1.18) | 0.32 |  |  |
| Age ≥ median (61 years) (N=256) and < median (N=258) | Age ≥ median (N=112/256, 43.8%)  Age < median (N=114/258, 44.2%) | 0.98 (0.69-1.39) | 0.93 |  |  |
| **Tumor types** |  |  |  |  |  |
| Colorectal (N=140)  Non colorectal (N=374) | Colorectal (N=61/140, 43.6%)  Non colorectal (N=165/374, 44.1%) | 0.98 (0.66-1.45) | 0.92 |  |  |
| Pancreatic (N=55)  Non pancreatic (N=459) | Pancreatic (N=23/55, 41.8%)  Non pancreatic (N=203/459, 44.2%) | 0.91 (0.51-1.60) | 0.78 |  |  |
| Breast (N=49)  Non breast (n=465) | Breast (N=19/49, 38.8%)  Non breast (N=207/465, 44.5%) | 0.79 (0.43-1.44) | 0.45 |  |  |
| Ovarian (N=43)  Non ovarian (N=471) | Ovarian (N=16/43, 37.2%)  Non ovarian (N=210/471, 44.6%) | 0.74 (0.39-1.40) | 0.42 |  |  |
| High TMB (N=33)***  Not high TMB (N=417) | High TMB (N=13/33, 39.4%)  Not high TMB (N=198/417, 47.5%) | 0.72 (0.35-1.48) | 0.47 |  |  |
| High PD-1 (N=93)*  Not high PD-1 (N=421) | High PD-1 (N=9/93, 9.7%)  Not high PD-1 (N=217/421, 51.4%) | 0.10 (0.05-0.21) | **<0.0001** | **0.0006** | **Low ICOS is independently associated with not-high PD-1, not-high PD-L1, not-high CTLA-4, and low ICOS ligand**  **(Not-high, <75^th^ percentile RNA rank; low, <25^th^ percentile RNA rank)** |
| High PD-L1 (N=67)*  Not high PD-L1 (N=447) | High PD-L1 (N=12/67, 17.9%)  Not high PD-L1 (N=214/447, 47.9%) | 0.24 (0.12-0.46) | **<0.0001** | **0.01** |  |
| High CTLA4 (N=87)*  Not High CTLA4 (N=427) | High CTLA4 (N=4/87, 4.6%)  Not high CTLA4 (N=222/427, 52.0%) | 0.04 (0.016-0.12) | **<0.0001** | **<0.0001** |  |
| High LAG-3 (N=116)*  Not High LAG-3 (N=398) | High LAG-3 (N=30/116, 25.9%)  Not high LAG-3 (N=196/398, 49.3%) | 0.36 (0.23-0.57) | **<0.0001** | 0.33 |  |
| High ICOSL(N=192)*  Not High ICOSL (N=322) | High ICOSL (N=75/192, 39.1%)  Not high ICOSL (N=151/322, 46.9%) | 0.73 (0.50-1.04) | 0.10 |  |  |
| Low ICOSL (N=76)*  Not Low ICOSL (N=438) | Low ICOSL (N=49/76, 64.5%)  Not low ICOSL (N=177/438, 40.4%) | 2.68 (1.61-4.44) | **0.0001** | **0.0007** |  |

* High CTLA4, High PD-1, High PD-L1, High LAG-3, High ICOSL ≥75 percentile RNA rank; Low ICOS, Low ICOSL 0-24 percentile RNA rank; Not High CTLA4, Not High PD-1, Not High PD-L1, Not High LAG-3, Not High ICOSL <75 percentile RNA rank; Not Low ICOS, Not Low ICOSL >24 percentile RNA rank

**p value that were significant (p ≤0.05) were included in multivariate analysis

*** High TMB, ≥10 mutations/Mb; Not high TMB <10 mutations/Mb

**Abbreviations:** ICOS= inducible T-cell co-stimulator; ICOSL= inducible T-cell co-stimulator ligand; TMB=tumor mutational burden

**Supplemental Table 2. Factors associated with high (>75^th^ percentile RNA rank) ICOS Ligand (ICOSL) expression**

|  | **High ICOS Ligand* (N=192)** | **Odds ratio (95% CI)** | **Univariate P value** | **Multivariate P value**** | **Comment** |
| --- | --- | --- | --- | --- | --- |
| Men (N=204)  Women (N=310) | Men (N=81/204, 39.7%)  Women (N=111/310, 35.8 %) | 1.18 (0.82-1.70) | 0.40 |  |  |
| Age ≥ median (61 years) (N=256) and < median (N=258) | Age ≥ median (N=96/256, 37.5%)  Age < median (N=96/258, 37.2%) | 1.01 (0.71-1.45) | 1.00 |  |  |
| **Tumor types** |  |  |  |  |  |
| Colorectal (N=140)  Non colorectal (N=374) | Colorectal (N=68/140, 48.6%)  Non colorectal (N=124/374, 33.2%) | 1.90 (1.28-2.83) | **0.0015** | **0.0012** | **High ICOSL is independently associated with colorectal cancer and with not-high PD-L1 and with high CTLA4** |
| Pancreatic (N=55)  Non pancreatic (N=459) | Pancreatic (N=24/55, 43.6%)  Non pancreatic (N=168/459, 36.6%) | 1.34 (0.76-2.36) | 0.31 |  |  |
| Breast (N=49)  Non breast (n=465) | Breast (N=17/49, 34.7%)  Non breast (N=175/465, 37.6%) | 0.88 (0.47-1.63) | 0.76 |  |  |
| Ovarian (N=43)  Non ovarian (N=471) | Ovarian (N=16/43, 37.2%)  Non ovarian (N=176/471, 37.4%) | 0.99 (0.52-1.89) | 1.00 |  |  |
| High TMB (N=33)***  Not high TMB (N=417) | High TMB (N=12/33, 36.4%)  Not high TMB (N=153/417, 36.7%) | 0.99 (0.47-2.06) | 1.00 |  |  |
| High PD-1 (N=93)*  Not high PD-1 (N=421) | High PD-1 (N=43/93, 46.2%)  Not high PD-1 (N=149/421, 35.4%) | 1.57 (1.00-2.47) | 0.058 |  |  |
| High PD-L1 (N=67)*  Not high PD-L1 (N=447) | High PD-L1 (N=17/67, 25.4%)  Not high PD-L1 (N=175/447, 39.2%) | 0.53 (0.30-0.95) | **0.03** | **0.003** |  |
| High CTLA4 (N=87)*  Not High CTLA4 (N=427) | High CTLA4 (N=45/87, 51.7%)  Not high CTLA4 (N=147/427, 34.4%) | 2.04 (1.28-3.25) | **0.0034** | **0.0001** |  |
| High LAG-3 (N=116)*  Not High LAG-3 (N=398) | High LAG-3 (N=41/116, 35.3%)  Not high LAG-3 (N=151/398, 37.9%) | 0.89 (0.58-1.38) | 0.66 |  |  |
| High ICOS (N=70)*  Not High ICOS (N=444) | High ICOS (N=29/70, 41.4%)  Not high ICOS (N=163/444, 36.7%) | 1.22 (0.73-2.04) | 0.51 |  |  |
| Low ICOS (N=226)*  Not Low ICOS (N=288) | Low ICOS (N=75/226, 33.2%)  Not low ICOS (N=117/288, 40.6%) | 0.73 (0.50-1.04) | 0.10 |  |  |

* High CTLA4, High PD-1, High PD-L1, High LAG-3, High ICOS ligand ≥75 percentile RNA rank; Low ICOS 0-24 percentile RNA rank; Not High CTLA4, Not High PD-1, Not High PD-L1, Not High LAG-3, Not High ICOS ligand <75 percentile RNA rank; Not Low ICOS >24 percentile RNA rank;

**p value that were significant (p ≤0.05) were included in multivariate analysis

***High TMB, ≥10 mutations/Mb; Not high TMB <10 mutations/Mb

Abbreviations: ICOS= inducible T-cell co-stimulator; ICOSL= inducible T-cell co-stimulator ligand; TMB=tumor mutational burden

**Supplemental Table 3. Factors associated with low (<25^th^ percentile RNA rank) ICOS Ligand (ICOSL) expression**

|  | **Low ICOS Ligand* (N=76)** | **Odds ratio (95% CI)** | **Univariate P value** | **Multivariate P value**** | **Comment** |
| --- | --- | --- | --- | --- | --- |
| Men (N=204)  Women (N=310) | Men (N=31/204, 15.2%)  Women (N=45/310, 14.5%) | 1.06 (0.64-1.73) | 0.90 |  |  |
| Age ≥ median (61 years) (N=256) and < median (N=258) | Age ≥ median (N=41/256, 16.0%)  Age < median (N=35/258, 13.6%) | 1.22 (0.75-1.98) | 0.46 |  |  |
| **Tumor types** |  |  |  |  | **Low ICOS ligand is independently associated with non-colorectal cancer and low ICOS** |
| Colorectal (N=140)  Non colorectal (N=374) | Colorectal (N=6/140, 4.3%)  Non colorectal (N=70/374, 18.7%) | 0.19 (0.08-0.46) | **<0.0001** | **0.0001** |  |
| Pancreatic (N=55)  Non pancreatic (N=459) | Pancreatic (N=8/55, 14.6%)  Non pancreatic (N=68/459, 14.8%) | 0.98 (0.44-2.16) | 1.00 |  |  |
| Breast (N=49)  Non breast (n=465) | Breast (N=5/49, 10.2%)  Non breast (N=71/465, 15.3%) | 0.63 (0.24-1.65) | 0.40 |  |  |
| Ovarian (N=43)  Non ovarian (N=471) | Ovarian (N=7/43, 16.3%)  Non ovarian (N=69/471, 14.7%) | 1.13 (0.48-2.65) | 0.82 |  |  |
| High TMB (N=33)***  Not high TMB (N=417) | High TMB (N=3/33, 9.1%)  Not high TMB (N=66/417, 15.8%) | 0.53 (0.16-1.79) | 0.45 |  |  |
| High PD-1 (N=93)*  Not high PD-1 (N=421) | High PD-1 (N=7/93, 7.5%)  Not high PD-1 (N=69/421, 16.4%) | 0.42 (0.18-0.94) | **0.03** | 0.46 |  |
| High PD-L1 (N=67)*  Not high PD-L1 (N=447) | High PD-L1 (N=13/67, 19.4%)  Not high PD-L1 (N=63/447, 14.1%) | 1.47 (0.76-2.84) | 0.27 |  |  |
| High CTLA4 (N=87)*  Not High CTLA4 (N=427) | High CTLA4 (N=6/87, 6.9%)  Not high CTLA4 (N=70/427, 16.4%) | 0.38 (0.16-0.90) | **0.02** | 0.38 |  |
| High LAG-3 (N=116)*  Not High LAG-3 (N=398) | High LAG-3 (N=20/116, 17.2%)  Not high LAG-3 (N=56/398, 14.1%) | 1.27 (0.73-2.22) | 0.46 |  |  |
| High ICOS (N=70)*  Not High ICOS (N=444) | High ICOS (N=9/70, 12.9%)  Not high ICOS (N=67/444, 15.1%) | 0.83 (0.39-1.75) | 0.72 |  |  |
| Low ICOS (N=226)*  Not Low ICOS (N=288) | Low ICOS (N=49/226, 21.7%)  Not low ICOS (N=27/288, 9.4%) | 2.68 (1.61-4.44) | **0.0001** | **0.0032** |  |

* High CTLA4, High PD-1, High PD-L1, High ICOS, High LAG-3 ≥75 percentile RNA rank; Low ICOS ligand 0-24 percentile RNA rank; Not High CTLA4, Not High PD-1, Not High PD-L1, Not High LAG-3, Not High ICOS <75 percentile RNA rank; Not Low ICOS ligand >24 percentile RNA rank

**p value that were significant (p ≤0.05) were included in multivariate analysis

*** High TMB, ≥10 mutations/Mb; Not high TMB <10 mutations/Mb

Abbreviations: ICOS= inducible T-cell co-stimulator; ICOSL= inducible T-cell co-stimulator ligand; TMB=tumor mutational burden

**Supplemental Table 4. Examples of clinical trials targeting ICOS in Cancer**

| **Therapy** | **Combination** | **Target** | **Phase** | **Cancer type** | **NCT number** |
| --- | --- | --- | --- | --- | --- |
| BMS-986226 | Ipilimumab,  Nivolumab | ICOS  (agonist) | 1/2 | Solid tumors | NCT03251924 |
| Feladilimab (GSK3359609) | Single agent,  Chemotherapy,  Pembrolizumab,  Dostarlimab,  Cobolimab  Bintrafusp alfa | ICOS  (agonist) | 1 | Advanced solid tumors | NCT02723955 |
|  | Immunotherapy | ICOS  (agonist) | 1 | Solid tumors | NCT03447314 |
|  | Pembrolizumab,  5FU-platinum | ICOS  (agonist) | 2/3 | Head and neck cancer | NCT04428333 |
|  | Tremelimumab or standard of care (chemo or cetuximab) | ICOS  (agonist) | 1/2 | Solid tumors | NCT03693612 |
|  | Pembrolizumab | ICOS  (agonist) | 2/3 | Head and neck cancer | NCT04128696 |
| Vopratelimab  (JTX-2011) | Anti-PD-1 or  Anti-CTLA-4 | ICOS  (agonist) | 1/2 | Refractory solid tumors | NCT02904226 |
|  | JTX-4014  (PD-1 inhibitor) | ICOS  (agonist) | 2 | NSCLC | NCT04549025 |
|  | Anti-PD-1 or  Anti-CTLA-4 | ICOS  (agonist) | 1/2 | Solid tumors | NCT04319224 |
|  | Anti-CTLA-4 | ICOS  (agonist) | 2 | NSCLC/urothelial cancer | NCT03989362 |
| XmAb23104 | Single agent,  Ipilimumab | ICOS  (agonist)/  anti-PD-1 | 1 | Solid tumors | NCT03752398 |
|  | XmAb22841 (CTLA-4 and LAG3) | ICOS (agonist)/ anti-PD-1 | 1/2 | Melanoma | NCT05695898 |
|  | Not applicable | ICOS (agonist)/  Anti-PD-1 | 2 | Advanced sarcoma | NCT05879185 |
| MEDI-570 | Not applicable | ICOS  (antagonist) | 1 | Peripheral T-cell lymphoma follicular variant and Angioimmunoblastic T-cell lymphoma | NCT02520791 |
| KY1044/  Alomfilimab SAR445256 | Single agent,  Atezolizumab | ICOS  (stimulates Teff with low ICOS/depletes Treg with high ICOS) | 1/2 | Advanced cancer | NCT03829501 |

**Abbreviations:** ICOS= inducible T cells co-stimulator, ORR=overall response rate, Teff=effector T-cell, Treg=regulatory T-cell


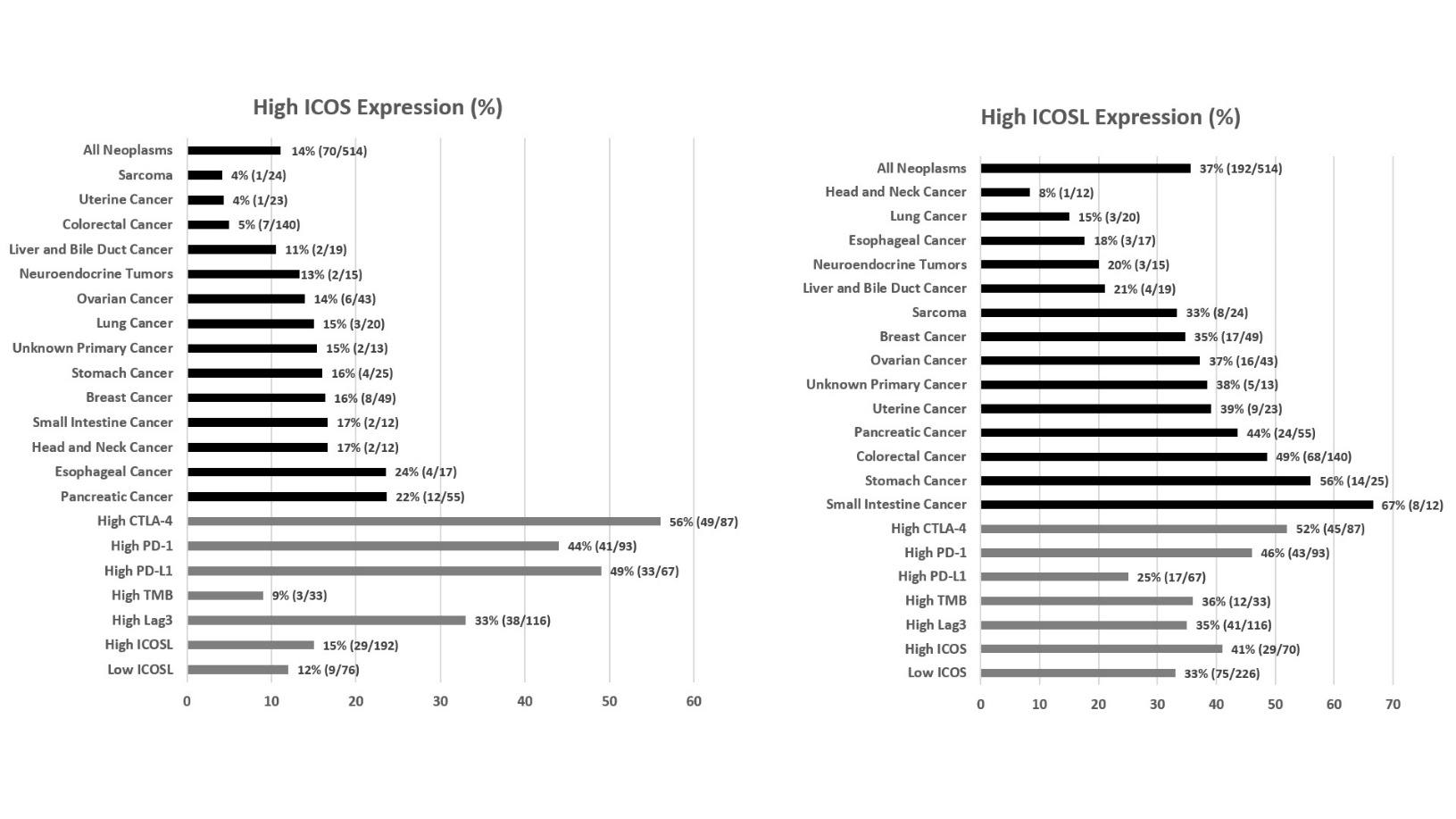


**Supplemental Figure 1A**


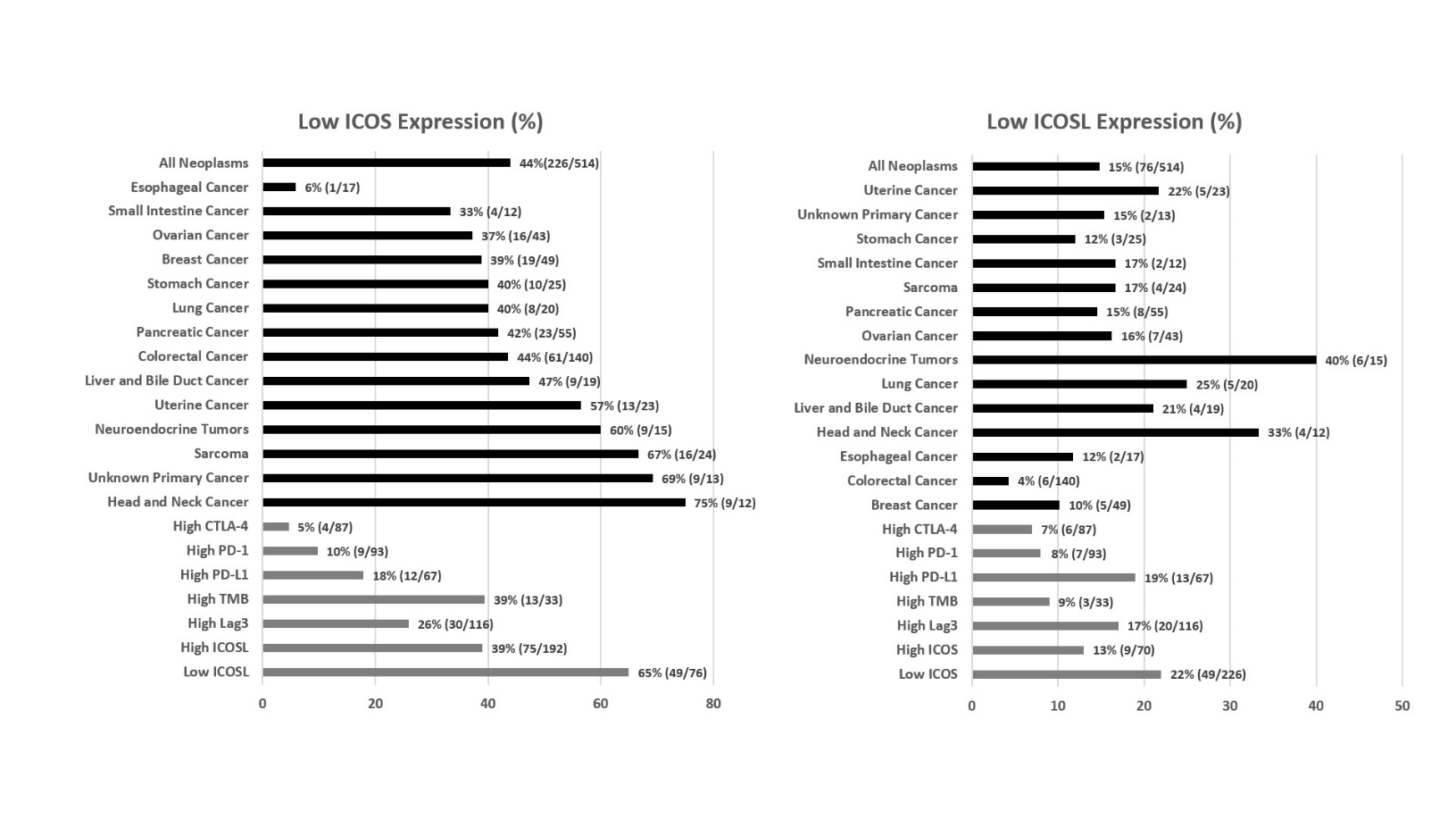


**Supplemental Figure 1B**

**Supplemental Figure 1**: ICOS and ICOSL expression according to cancer type. A. High ICOS and ICOSL expression. B. Low ICOS and ICOSL expression. High expression, ≥75 percentile RNA rank and low expression, 0-24 percentile RNA rank. Only cancers with at least 10 samples were included in the figure. Abbreviations: ICOS= inducible T-cell co-stimulator; ICOSL= inducible T-cell co-stimulator ligand; TMB=tumor mutational burden


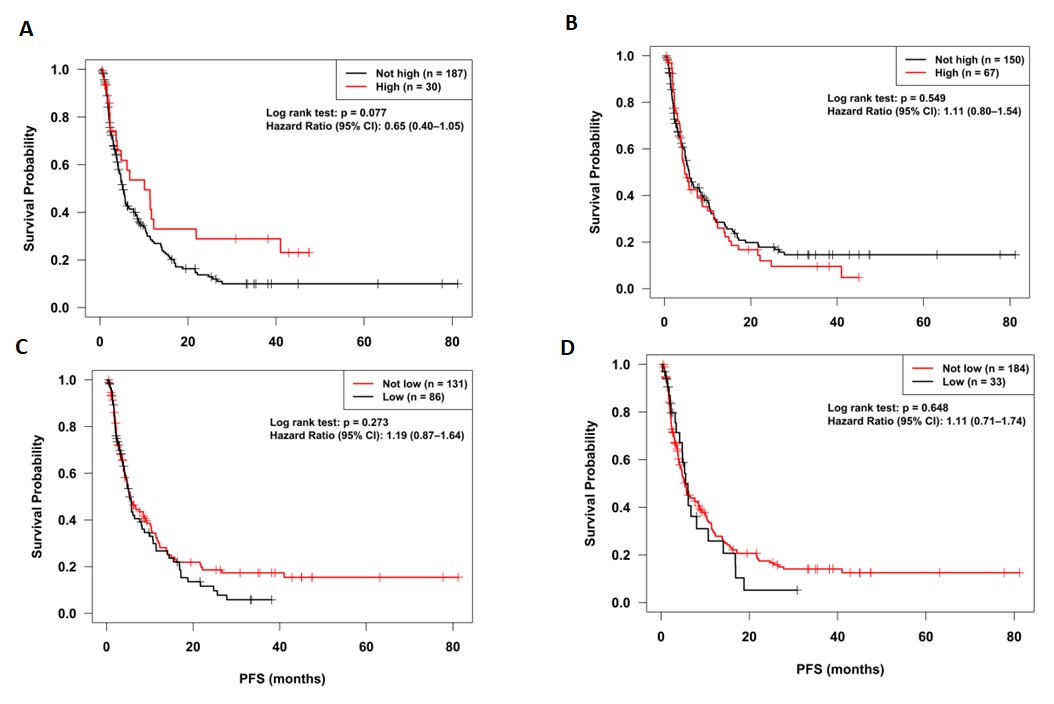


**Supplemental Figure 2**: Progression-free survival (PFS) for patients who received immunotherapy from the start date of immunotherapy. A. ICOS high vs. not high. B. ICOS ligand high vs. not high. C. ICOS low vs. not low. D. ICOS ligand low vs. not low. High ICOS, high ICOS ligand ≥ 75 percentile RNA rank; Low ICOS and ICOS ligand 0-24 percentile RNA rank**.**


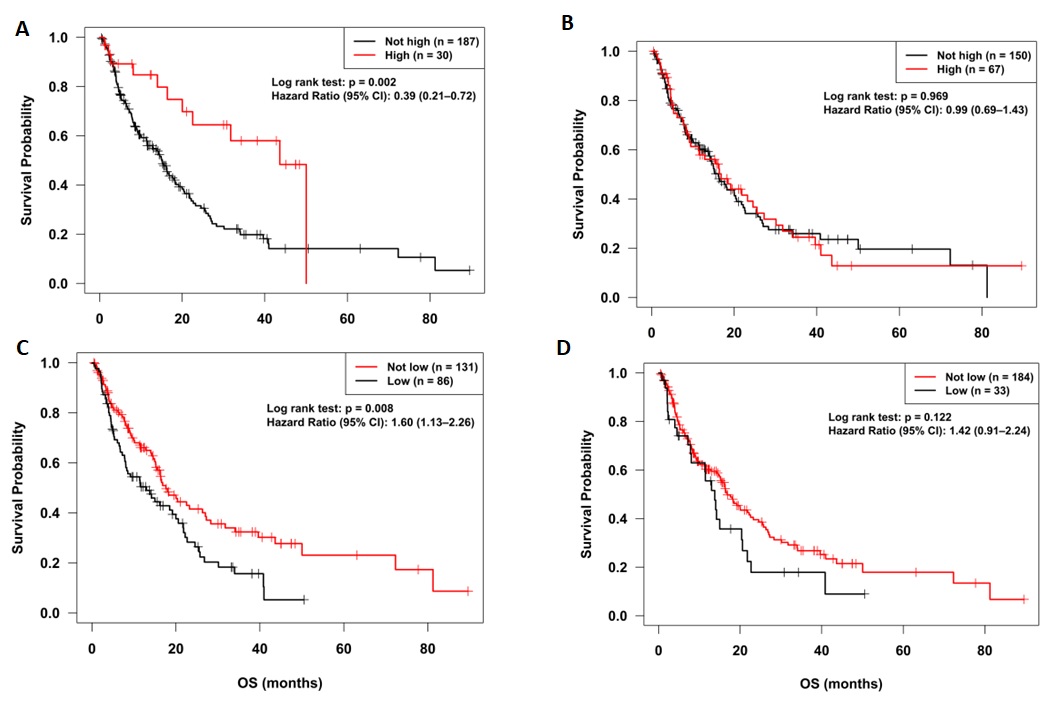


**Supplemental Figure 3**: Overall survival (OS) for patients who received immunotherapy from the start date of immunotherapy. A. ICOS high vs. not high. B. ICOS ligand high vs. not high. C. ICOS low vs. not low. D. ICOS ligand low vs. not low. High ICOS, high ICOS ligand ≥ 75 percentile RNA rank; Low ICOS and ICOS ligand 0-24 percentile RNA rank.


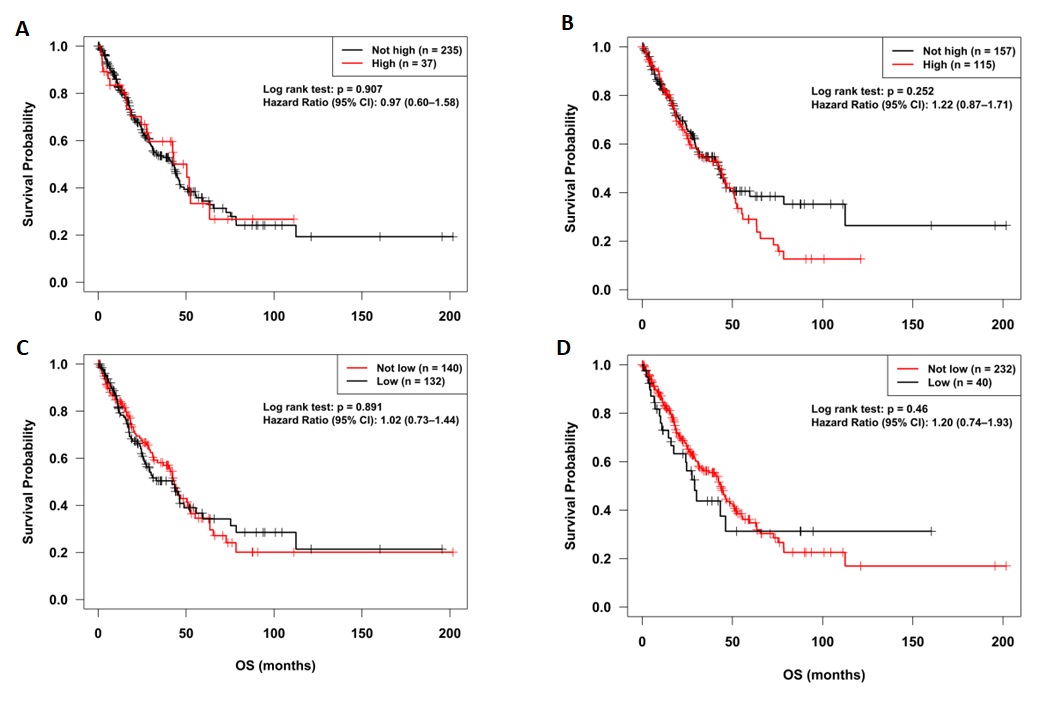


**Supplemental Figure 4**: Overall survival (OS) for patients not receiving immunotherapy from the date of diagnosis of advanced or metastatic disease. A. ICOS high vs. not high. B. ICOS ligand high vs. not high. C. ICOS low vs. not low. D. ICOS ligand low vs. not low. High ICOS, high ICOS ligand ≥ 75 percentile RNA rank; Low ICOS and ICOS ligand 0-24 percentile RNA rank.
